# Data Resource Profile: Linking nationwide health and social registries in Estonia (BIG-HEART)

**DOI:** 10.1093/ije/dyag027

**Published:** 2026-03-09

**Authors:** Laura Lõo, Nikita Umov, Marek Oja, Sulev Reisberg, Anneli Uusküla, Raivo Kolde, Taavi Tillmann

**Affiliations:** Institute of Family Medicine and Public Health, University of Tartu, Tartu, Estonia; Institute of Family Medicine and Public Health, University of Tartu, Tartu, Estonia; Institute of Computer Science, University of Tartu, Tartu, Estonia; Institute of Computer Science, University of Tartu, Tartu, Estonia; STACC, Tartu, Estonia; Institute of Family Medicine and Public Health, University of Tartu, Tartu, Estonia; Institute of Computer Science, University of Tartu, Tartu, Estonia; Institute of Family Medicine and Public Health, University of Tartu, Tartu, Estonia

**Keywords:** cardiovascular disease, health inequalities, data linkage, nationwide cohort, social determinants of health, administrative data, electronic health records, Observational Medical Outcomes Partnership Common Data Model

Key Features

- The BIG-HEART cohort was established to study and reduce health inequalities in cardiovascular disease by linking rich, multidimensional electronic health and social data across Estonia.
- The dataset includes all individuals aged ≥36 years residing in Estonia in 2012 (*N*= 770 323). Its full population coverage minimizes sampling and healthy-volunteer bias. Existing funding and permits will support annual health outcome follow-up through to at least 2026, with possible future extensions.
- The dataset integrates all routinely collected individual-level primary and secondary care health data (including in- and outpatient visits, diagnoses, prescriptions issued and filled), mortality data, and extensive social data (e.g. ethnicity, education, marital status, social benefits, unemployment history, land and business ownership) from eight national registries. This enables the exploration of novel social epidemiology dimensions—such as unbiased wealth measures, medication adherence, and care quality—and supports the development of equity-enhancing clinical risk-prediction algorithms and large language models.
- Health and social data are linked by using anonymized patient identifiers that are generated from national personal identification numbers, ensuring both accuracy and privacy. The health data are stored in the Observational Medical Outcomes Partnership Common Data Model, facilitating international collaboration.
- Collaboration inquiries are welcome and can be directed to the BIG-HEART team at taavi.tillmann@ut.ee.

## Data resource basics

### The rationale for establishing BIG-HEART

Cardiovascular disease (CVD) remains a leading global public health concern despite being largely preventable [1, 2]. Sixty years of research has shown a strong association between socio-economic factors and CVD risk [3]. However, these insights have rarely been integrated into clinical practice. Primary and secondary prevention services can exacerbate health inequalities when attendance at screening programs, preventive medication use, and success with behaviour change services follow social gradients [4–6]. We propose that integrating multidimensional socio-economic and psychosocial data with electronic health records can, first, deepen our understanding of the drivers and patterns of health inequalities. Second, it can accelerate implementation by enabling the developers of risk-prediction algorithms and care services to directly incorporate socio-economic factors into their models and pathways. The existing CVD risk-prediction models recommended in clinical practice [7–9] typically incorporate at most one socio-economic indicator, despite emerging evidence suggesting that each additional layer may enhance both predictive performance and fairness [10, 11]. This lack of integration may worsen inequalities from the earliest stage of care—risk prediction—compounded by unequal uptake and adherence to recommended services [5, 12–14]. Estonia has among the highest CVD mortality rates in Europe—nearly twice the EU average—and modestly above the global average, which likely reflects a higher disease incidence rather than poorer outcomes after diagnosis [15–18]. This underscores both the statistical power and the public health potential of conducting CVD research in Estonia. BIG-HEART integrates health and social data to bridge basic epidemiological research with applied solutions, promoting more precise and equitable risk assessments and services.

### Estonian health and social data

Estonia—a country in north-eastern Europe with a population of 1.3 million—has long led the way globally in building secure and efficient digital infrastructures for public services. Since 1989, every resident has been assigned an 11-digit personal identification code, facilitating data linkage across government systems. In 2001, Estonia launched ‘X-Road’—a secure data-exchange system enabling interoperability across governmental databases while maintaining strict security standards [19]. Managed by the Estonian Information System Authority, this infrastructure supports resilience to changes in underlying database architectures or digital services.

As of 2023, ∼95% of Estonia’s population was covered by the publicly funded Estonian Health Insurance Fund (EHIF) [20], which is similar to the UK’s National Health Service [21, 22]. Estonia’s healthcare system includes roughly 400 primary care centres [23] and 20 hospitals [24], each with its own information technology system. These local systems are not fully interoperable, allowing implementation flexibility. However, providers must submit claims, prescriptions, and discharge summaries to the national system, enabling comprehensive, standardized coverage. Estonia’s e-prescribing platform records all prescriptions and dispensing statuses, supporting the assessment of initiation, adherence, and persistence. Blockchain technology logs all clinician access to personal health data, enhancing transparency and trust. Residents can access their records via the national Health Portal [25], which is similar to systems in Finland and Iceland [26].

### Secondary use of Estonian health data for research

As the collection and coding of electronic health data differ across countries, there is growing interest in harmonizing cleaned, research-ready datasets rather than raw data. The Observational Medical Outcomes Partnership (OMOP) Common Data Model (CDM) [27] has become a widely adopted standard for enabling cross-country comparisons and large-scale analyses. Estonia contributed early to this effort, first mapping a 10% random sample of national health data to OMOP CDM [28], followed by a sample that supported several international COVID-19 studies. These analyses provided valuable insights—e.g. they confirmed that post-acute COVID-19 symptoms defined by the World Health Organization were frequently reported, though with variation across healthcare settings and countries [29]. They also demonstrated that vaccination, particularly the first dose, reduced the risk of long Covid [30], and showed that serious adverse events such as thromboembolism and myocarditis were more common after SARS-CoV-2 infection than in historical cohorts [31].

### BIG-HEART: advancing health and social data integration

Building on Estonia’s electronic health record infrastructure, BIG-HEART further integrates data by enriching health records with socio-economic data. BIG-HEART was established in 2024, covering all 770 323 Estonian residents aged ≥36 years who, as of 1 January 2012, were resident in Estonia according to the Population Register. This fixed cohort is tracked annually for health and mortality outcomes through to 2026. All health data have been transformed into the OMOP CDM, supporting harmonized, large-scale analyses and international collaborations. Further information on the data-transformation process and data-quality assessments are provided in the Supplementary materials.

## Data collected

### Data sources

**Figure 1:**
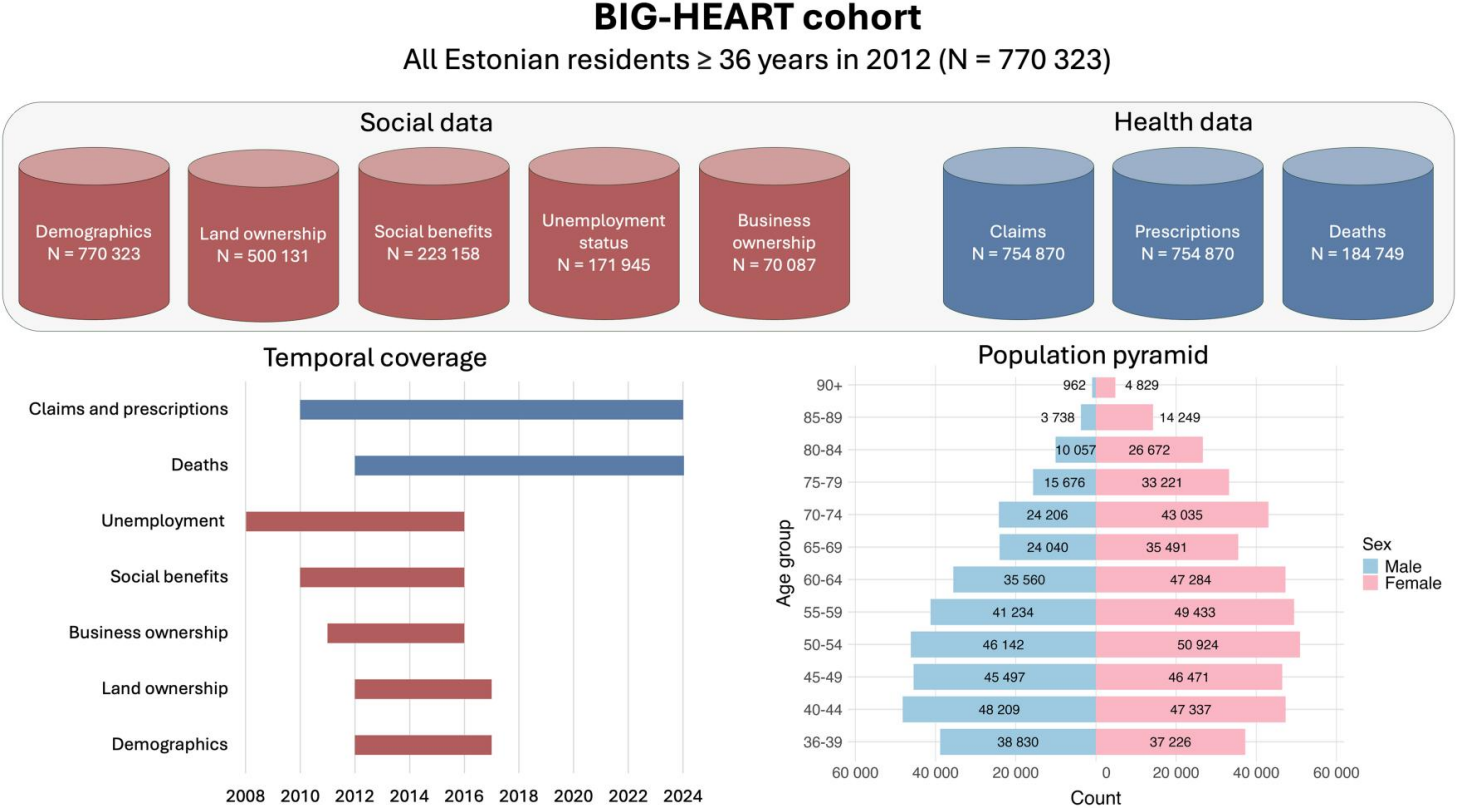
Data sources included in the data linkage, data timeliness, and population pyramid of the BIG-HEART cohort.

BIG-HEART is both a longitudinal fixed cohort and a data resource, enabling the analysis of health and social exposures over time. It is based on routinely collected, secondary administrative data, structured as a register-based cohort with repeated annual updates. The cohort was constructed by using the Population Register, which defined the study population. Table 1 shows the baseline demographic characteristics. Data are grouped into (i) health and (ii) social domains. Eight national datasets were linked at the individual level by using project-specific anonymized patient identifiers. Figure 1 provides an overview of the linked data sources, coverage, and age distribution.

**Table 1:** Baseline demographic characteristics of the BIG-HEART cohort (*N* = 770 323) as of 1 January 2012, from the Population Register.

Characteristic	Number of people	%
Age		
Mean (SD, range)	57.4	14, 36–108
Median (IQR)	56	46–68
Male sex	334 151	43.4
Citizenship		
Estonian	608 990	79.1
Russian	79 847	10.4
Stateless^a^	62 257	8.1
Ukrainian	4723	0.6
Finnish	4183	0.5
Latvian	1607	0.2
Lithuanian	1124	0.1
Other	7501	1.0
Missing	91	0.01
Self-reported nationality		
Estonian	499 090	64.8
Russian	204 487	26.5
Ukrainian	20 563	2.7
Finnish	8055	1.0
Latvian	1679	0.2
Lithuanian	1468	0.2
Other	28 282	3.7
Missing	6699	0.9
Education level^b^		
Less than primary	3389	0.4
Primary (age ∼11 years)	34 198	4.4
Lower secondary (age ∼15 years)	94 820	12.3
Lower secondary, vocational	15 564	2.0
Upper secondary (age ∼18 years)	192 709	25.0
Upper secondary, vocational	73 164	9.5
Upper secondary, specialized	141 479	18.4
Bachelor’s degree	37 645	4.9
Master’s degree	126 368	16.4
Doctorate	4904	0.6
Missing	46 083	6.0
Marital status		
Married	379 490	49.3
Divorced	155 869	20.2
Widow/widower	100 471	13.0
Single	111 964	14.5
Missing	22 529	2.9

IQR, interquartile range.

a ‘Stateless’ denotes persons with stateless or undetermined citizenship. Many hold an Estonian Alien’s Passport—an identity and travel document issued to legally resident individuals who do not possess the citizenship of any state. Historically, this group formed after the restoration of Estonian independence in 1991, when citizenship was restored to pre-1940 citizens and their descendants. Residents who lacked provable pre-war ties and who did not obtain citizenship through naturalization remained without citizenship. This group has steadily decreased over the decades.

b Education levels and associated ages are aligned with the Soviet-era school structure, as the youngest individuals in the dataset were born prior to Estonia’s post-independence educational reforms. Certain categories (such as upper secondary specialized) have since been discontinued in the modern system.

‘Health data’ are primarily derived from a national billing database managed by the EHIF [32]. This comprehensive dataset includes all reimbursed insurance claims submitted by primary and secondary healthcare providers, covering both in- and outpatient services. Each claim records the procedures performed and diagnoses—both incident and pre-existing—coded according to the International Statistical Classification of Diseases, 10th Revision (ICD-10), and the Nordic Medico-Statistical Committee (NOMESCO) Classification of Surgical Procedures, as well as local service codes used in the national health insurance system. Table 2 highlights key public health diseases by gender, based on the top 30 conditions ranked by disability-adjusted life years and other commonly reported cases from the BIG-HEART database.

**Table 2:** Number and percentage of participants with selected diseases of significant public health impact, based on disability-adjusted-life-year rankings and commonly reported conditions, in the BIG-HEART cohort (*N* = 770 323). Results are shown overall and stratified by gender, including individuals with prevalent disease at baseline or incident disease during follow-up.

Diagnosis	ICD-10 code^a^	Total (%)	Male (%)	Female (%)
		*N* = 770 323	*N* = 334 151	*N* = 436 172
Hypertensive heart disease	I10–15	497 689 (64.6)	206 281 (61.7)	291 408 (66.8)
Dorsalgia	M54	432 389 (56.1)	168 473 (21.9)	263 916 (60.5)
Dyslipidaemia	E78	337 548 (43.8)	130 040 (38.9)	207 548 (47.6)
Falls	W00–19	314 613 (40.8)	127 337 (38.1)	187 276 (42)
COVID-19	U07	270 700 (35.1)	105 582 (31.6)	165 118 (37.9)
Virus identified	U07.1	176 531 (22.9)	70 645 (21.1)	105 886 (24.3)
Virus not identified	U07.2	139 851 (18.2)	50 383 (15.1)	89 468 (20.5)
Heart failure	I50	205 515 (26.7)	81 051 (24.3)	124 464 (28.5)
Ischaemic heart disease	I21–25	149 556 (19.4)	69 703 (20.9)	79 853 (18.3)
Type 2 diabetes	E11	131 648 (17.1)	54 188 (16.2)	77 460 (17.8)
Cerebrovascular disease	I60–69	129 454 (16.8)	49 227 (14.7)	80 227 (18.4)
Depression	F32–33	137 495 (17.8)	38 018 (11.4)	99 477 (22.8)
Anxiety	F41	104 126 (13.5)	25 928 (7.8)	78 198 (17.9)
Asthma	J45	83 794 (10.9)	29 378 (8.8)	54 415 (12.5)
Chronic obstructive pulmonary disease	J44	72 460 (9.4)	39 687 (11.9)	32 773 (7.5)
Chronic kidney disease	N18	64 148 (8.3)	25 578 (7.7)	38 570 (8.8)
Age-related hearing loss	H91	58 756 (7.6)	21 564 (6.5)	37 182 (8.5)
Prostate cancer	C61	22 946 (3.0)	22 946 (6.9)	N/A^b^
Migraine	G43	24 296 (3.2)	3 554 (1.1)	20 742 (4.8)
Colorectal cancer	C18–21	18 754 (2.4)	8389 (2.5)	10 365 (2.4)
Breast cancer	C50	17 208 (2.2)	N/A	17 208 (3.9)
Psychotic disorders	F20–25, F28–29	15 366 (2.0)	5490 (1.6)	9876 (2.3)
Type 1 diabetes	E10	14 810 (1.9)	6720 (2.0)	8090 (1.8)
Lung cancer	C34	13 431 (1.7)	9107 (2.7)	4324 (1.0)
Alcohol addiction or use of specialist services	Z71.4, Z72.1, F10	46 548 (6.0)	36 150 (10.8)	10 398 (2.4)
Tobacco addiction or use of cessation services	Z71.6, Z72.0, F17	13 532 (1.8)	8063 (2.4)	5469 (1.3)
Other COVID-19 outcomes	U09	6237 (0.8)	2171 (0.6)	4066 (0.9)
Pancreatic cancer	C25	4799 (0.6)	2155 (0.6)	2644 (0.6)
Use of psychoactive substances	F11–16, F18–19	4472 (0.6)	1836 (0.5)	2636 (0.6)
Cervical cancer	C53	3747 (0.5)	N/A	3747 (0.9)
Alzheimer’s disease	G30	3229 (0.4)	896 (0.3)	2333 (0.5)
Manic episode	F31	2577 (0.3)	878 (0.3)	1699 (0.4)
COVID-19 vaccine causing adverse effects	U12	807 (0.1)	234 (0.1)	573 (0.1)
Multisystem inflammatory syndrome associated with COVID-19	U10	357 (0.05)	132 (0.04)	225 (0.1)
Bipolar affective disorder	F30	495 (0.1)	186 (0.1)	309 (0.1)

a ICD-10, the International Classification of Diseases, 10th Revision.

b N/A indicates diagnoses not applicable to this sex or with uncertain classification.

The EHIF database contains comprehensive information on sick-leave episodes and health insurance coverage, including the start and end dates of coverage and the reasons behind changes in status. Our data suggest that 6%–8% of individuals may experience fluctuations in their insurance coverage, implying that stable coverage is closer to 91%, rather than the usually reported 95%. These fluctuations may be due to changes in employment or registration status and should be taken into account when interpreting analyses based on insurance data. In addition, the EHIF also maintains an extensive prescription records system, with detailed information on all dispensed medications—classified by using the Anatomical Therapeutic Chemical system—along with the prescribed dosage, prescribing provider, and both the date and location of dispensing. Metadata from packaging codes further enrich these records by identifying the drug manufacturer, strength, and pharmaceutical form (e.g. tablet, cream).

The Causes of Death Register [33] collects comprehensive mortality data. In the BIG-HEART cohort, we have full details on the cause of death when the underlying cause is circulatory (ICD-10 codes I00–I99) or sudden death of unknown cause (R96.0, R96.1). For all other causes, the date of death is recorded, which supports time-to-event analyses and the handling of competing non-cardiovascular outcomes. Should future research require detailed cause-of-death data beyond the circulatory domain, extended access can be requested to support scientifically justified hypotheses.

‘Social data’ are currently available from five registries. Table 3 presents the socio-economic and health-related characteristics of the BIG-HEART cohort across the study period. While the primary purpose of collecting social data was to characterize the baseline exposures, future extensions may incorporate variables such as occupation and income if supported by research hypotheses. Consequently, social data have shorter time coverage than health data. The Population Register [34] includes demographic variables such as sex, birth year, marital status, citizenship, self-reported nationality, county of residence, emigration date, and death date, as well as the most recently recorded educational attainment. Citizenship refers to a person’s legal citizenship as recognized by the state, whereas self-reported nationality reflects ethnic or cultural identity as declared by the individual. Citizenship categories include ‘Stateless’—a unique group of legally resident individuals without any state citizenship (see Table 1). As education data were provided as a 2023 snapshot rather than time-stamped records, we cannot determine participants’ education status at the baseline and assume no changes during follow-up.

**Table 3:** Socio-economic and health characteristics of the BIG-HEART cohort (*N* = 770 323) over the full study period, from linked databases.

Data source and temporal coverage	Characteristic	Number of people (%)
Social Insurance Records Database 2010–16	Receiving any social benefit at least once between 2010 and 2016	223 158 (29.0)
	Benefit for having a disability	164 940 (21.4)
	Lifelong pension for having no capacity to work	105 951 (13.8)
	Victim of Soviet political repression during 1945–91	11 874 (1.5)
	Occupational injury/disease	2363 (0.3)
	Forced to work during the Chernobyl clean-up in 1986–9	1464 (0.2)
E-Business Register 2011–16	**If analysing people as the unit of analysis**	
	Number of companies owned by one participant, at baseline	
	0	730 726 (94.9)
	1	32 631 (4.2)
	2	5057 (0.7)
	3	1250 (0.2)
	> 3	659 (0.1)
	**If analysing companies as the unit of analysis, at baseline**	
	Number of companies based on their size	
	Micro-enterprises (0–9 employees)	40 753
	Small enterprises (10–49 employees)	3142
	Medium and large enterprises (≥50 employees)	512
	Missing	34
	Average company’s staff size	
	Median	1
	Mean	5.6
	Median profit (euros, €)	742
	Median accumulated profit prior to baseline (€)	5894
	Dividends paid (€)	
	Median	5112
	Mean	35 389
	Number of loss-making companies	13 975
	Number of companies with accumulated losses	7374
E-Land Register 2012–17	Property ownership	500 131 (64.9)
	Owners of only non-residential property	96 826 (19.4)
	Owners of some residential property	403 305 (52.4)
	Owners of residential property in a major city^a^	232 355 (30.2)
	Owners of residential property in the capital city^b^	153 156 (19.9)
Unemployment Insurance Fund 2008–16	**If analysing people as the unit of analysis**	
	Unemployed at least once between 2008 and 2016	171 945 (22.3)
	Unemployed at least twice between 2008 and 2016	72 891 (9.5)
	**If analysing all unemployment events (*N* = 319 338) as the unit of analysis**	
	Median unemployment period in days (IQR) per episode	181 (76–337)
	Median time in days from finding a job until becoming unemployed again (IQR)	149 (62–297)
	Reason for registering as ‘unemployed’, top 3 reasons	
	Redundancy	41 781 (13.1)
	End of fixed-term contract	35 059 (11.0)
	Employee’s initiative	20 403 (6.4)
	Reason for terminating the ‘unemployed’ status, top 3 reasons	
	New employment	189 006 (59.2)
	Breach of unemployment obligations	80 085 (25.1)
	Requested by unemployed person	39 454 (12.4)
Estonian Health Insurance Fund 2010–24	People covered by the national health insurance system	
	Insured at least once between 2012 and 2023	754 870 (98.0)
	Insured at baseline (1 January 2012)	703 433 (91.3)
	Individuals with ≥90 days of sick leave	29 798 (3.9)
Death Register 2012–24	All-cause deaths between 2012 and 2023	184 749 (24.0)
	CVD deaths (ICD-10 codes I00–99) between 2012 and 2023	95 019 (12.3)
	Atherosclerotic death (ICD-10 codes I21–25 + I60–69) between 2012 and 2023	45 771 (5.9)

a Tallinn, Tartu, Narva, or Pärnu.

b Tallinn.

The Social Insurance Records Database [35] covers social benefits—such as type, amount, and payment period—for various reasons, including having a disability, being a victim of severe violence, political repression during the Soviet Union from 1945 to 1991, being forced to work during the Chernobyl clean-up operation from 1986 to 1989, living alone as a pensioner, suffering health damage from an occupational disease or work accident, or receiving an incapacity pension. This data are available from 2010 to 2016.

The Unemployment Insurance Fund data source [36] provides longitudinal data on unemployment episodes, prior occupations, and reasons for entering and exiting unemployment. For the ∼25% of participants with at least one unemployment spell, it is possible to reconstruct the pre-unemployment income, enabling nuanced analyses of economic vulnerability. Occupations are coded by using the International Standard Classification of Occupations, allowing longitudinal tracking of employment stability and career disruption. Evidence suggests that the frequency of labour-market transitions may be more predictive of disease incidence than unemployment duration [37]. Among the 10% of participants with multiple unemployment episodes, employment and unemployment often alternate in 6-month cycles. The BIG-HEART cohort enables investigation of the contextual and personal factors influencing unemployment resolution—whether through re-employment, retirement, or other paths. Currently, the cohort includes 8 years of employment data, which could be extended to 16 years in future updates if needed.

Objective wealth indicators are available from two registries. The E-Land Register [38] records all landholdings—residential or commercial—owned by participants, down to the neighbourhood level (excluding street names). As shown in Table 2, ∼65% of the BIG-HEART cohort owns some form of land, enabling rare analyses of how real-estate wealth relates to life expectancy, healthcare use, and outcomes—supporting fairness assessments across socio-economic strata.

The E-Business Register [39] provides data on ∼40 000 individuals who co-own or operate a legal entity. It offers 6 years of detailed longitudinal information, including turnover, profitability, tax arrears, employee count, and sector classification using Nomenclature of Economic Activities codes. Among the broader population, a smaller share (∼5%) owns business assets, typically through micro-enterprises. This supports research on how financial stability or instability in business ownership affects physical and mental health, healthcare use, and outcomes. Wealth across property and business domains offers a multidimensional view of financial security, precarious employment, and health—especially relevant during external shocks such as the COVID-19 pandemic. The dataset could be extended to examine whether business disruptions led to differences in healthcare access, diagnoses, or medication use.

### Secure data processing

To protect privacy, the Population Register assigned a unique, project-specific anonymized patient identifier to each person, replacing national personal identification numbers. These identifiers were shared with data providers to enable secure record linkage, but not with the University of Tartu, which never had access to identifiable information. Each data provider used the identifier to extract relevant records and returned anonymized datasets to a secure computing environment, ensuring that researchers only accessed de-identified data. More information on the secure computing environment can be found in the Supplementary materials.

## Data resource use

The BIG-HEART dataset became available to us in mid-2024, making it a new and highly promising resource for studying the socio-economic dimensions of health and healthcare. Although no studies have yet been published, several have been initiated by our small team. Among the healthy participants at the baseline, we are investigating whether the registry-based measures of real-estate and business wealth, unemployment, and social benefits predict CVD incidence and improve CVD risk-prediction algorithms. We are also assessing whether machine-learning, artificial intelligence, and large language models can outperform conventional regression models in accuracy and fairness—questions that the BIG-HEART cohort is exceptionally well placed to evaluate, with its rich socio-economic detail and complete population representation. This work informs the design of future interventions, such as identifying persons most amenable to preventive care (e.g. statins, antihypertensives, or behavioural services) and evaluating their effectiveness.

For persons with established disease at the baseline, we are describing the socio-demographic differences in healthcare use, including screening attendance and medication adherence, and developing models to predict future non-adherence, with potential feedback to clinicians. Beyond these initial studies, BIG-HEART is well positioned to address broader public health questions spanning computer science, epidemiology, and social sciences. We welcome collaborations to either analyse the existing data (typically yielding results within 3–6 months) or add new data layers via Estonian regulators (which take ∼12 months).

## Strengths and weaknesses

BIG-HEART has three core strengths. First, it includes a complete population sample: all Estonian residents aged ≥36 years were included via the Population Register. This eliminates sampling bias and sets BIG-HEART apart from biobanks and survey-based cohorts, in which participation is voluntary and often limited. Second, it offers complete healthcare service capture, due to Estonia’s national electronic infrastructure and OMOP-standardized data. These features support the accurate estimation of healthcare use and of conditions that usually result in clinical contact. Third, BIG-HEART integrates rich social data at the population level. These data, though not originally collected for research, are nearly complete and enable the rigorous analysis of social determinants. Social epidemiologists can evaluate how socio-economic factors affect disease, while data scientists can validate prediction models and assess fairness across subgroups.

Despite these strengths, BIG-HEART has limitations. First, some valuable data layers remain unlinked, including the Health Information System (with discharge summaries, clinical notes, and lab results), the Estonian Biobank (which has standardized its data to the OMOP CDM), Estonian Tax Board registries (with declared income and occupation for those never unemployed), and disease-specific registries for myocardial infarction and cancer. Second, the presented health needs may underestimate the true needs. Chronic conditions often precede care seeking and may go undocumented, particularly among disadvantaged groups, potentially biasing the risk estimates and fairness evaluations toward the null. Although official coverage is 95%, baseline data show that only 91% were insured at linkage and 6%–8% cycle in and out of coverage. If uninsured individuals have greater health needs, then the prevalence and fairness measures may be overly optimistic. Third, some individuals may have emigrated before the baseline or during follow-up without updating their residency status, leading to potential misclassification of exposure time. BIG-HEART is a fixed cohort and, while emigration data are available up to 2024, immigration data are not captured. Consequently, individuals who have left Estonia may remain classified as residents, whereas new immigrants after the baseline are not represented. Migration among adults in Estonia has been modest, suggesting that this limitation is unlikely to have substantially affected the representativeness. In addition, Estonia’s strong incentives to update residency information (e.g. for childcare and mortgage benefits) likely reduce the under-reporting of emigration. Future work may identify likely emigrants through disengagement from health and social systems.

## Data resource access

Access to the BIG-HEART data can be obtained with approval from the Research Ethics Committee of the University of Tartu. Researchers can contact the principal investigator Taavi Tillmann through taavi.tillmann@ut.ee.

## Ethics Approval

The BIG-HEART project has received approval from the Research Ethics Committee of the University of Tartu (no. 384/T-8, 20.11.2023).

## Supplementary Material

dyag027_Supplementary_Data

## Data Availability

See ‘Data resource access’ above.
