# Supplementary material for: Data Resource Profile: Linking nationwide health and social registries in Estonia (BIG-HEART)
Source: Int J Epidemiol. 2026 Mar 9;55(2):dyag027. doi: 10.1093/ije/dyag027 (PMC13016937; doi:10.1093/ije/dyag027)
Supplement: dyag027_Supplementary_Data [file dyag027_supplementary_data.docx]

## **OMOP Common Data Model**

The Observational Medical Outcomes Partnership Common Data Model (OMOP CDM) is an open standard maintained by the Observational Health Data Sciences and Informatics (OHDSI) community [1, 2]. It provides a harmonised database structure, a standardised vocabulary of clinical concepts, and a set of open-source analytic tools that enable reproducible research across heterogeneous healthcare data sources.

In the BIG-HEART project, all **health data** was transformed into the OMOP CDM (version 5.4.2) [3], enabling interoperability with international research networks. The transformed health data included health insurance claims, prescription records, and death registry information. The Extract-Transform-Load (ETL) process used for this transformation builds directly on the validated pipeline developed for a 10% national sample described by Oja *et al* (2023) [4]. Applying this established process ensured consistency with earlier Estonian implementations and alignment with OHDSI conventions. Socioeconomic data have not yet been transformed into the OMOP CDM.

Transforming the BIG-HEART health data to OMOP CDM provides benefits at multiple levels. Locally, researchers can use the extensive OHDSI tools for cohort definition, characterisation, and prediction tasks without building bespoke software. Nationally, the standardisation facilitates reuse and transportability of analytic definitions and models across Estonian databases. Internationally, BIG-HEART can now participate as a data partner in OHDSI federated studies or replicate findings from other OMOP data sources to validate results in the Estonian population.

## **DataQualityDashboard**

Data quality of the transformed BIG-HEART **health** data was assessed using the OHDSI *DataQualityDashboard* (version 2.6.3) [5]. This tool conducts automated validation and verification checks across the OMOP CDM, assessing conformance, completeness, and plausibility at the concept, field, and table levels. Identified issues were reviewed and corrected where feasible. Of the 2 342 checks performed, 2 294 passed and 48 failed. Of the 48 failed checks, 35 were related to cohort and cohort definition issues. 4 errors involved diagnoses assigned to persons with an incompatible gender (e.g., neoplasm of male genital organs assigned to a female). 9 errors were related to completeness tests, where certain tables lacked full mappings of source codes to standard concepts or where some patient information was not available (e.g., in the BIG-HEART, death cause is recorded only for cardiovascular events). Overall, the dataset met the expected quality standards. Supplementary Table S1 summarises the *DataQualityDashboard* results for the BIG-HEART health data mapped to OMOP CDM.


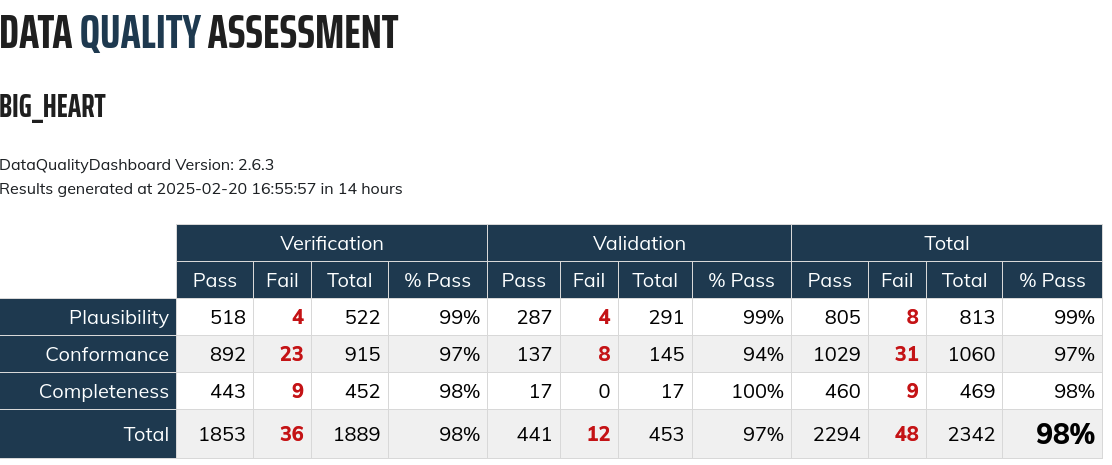


Supplementary Table S1. *DataQualityDashboard* results of the data transformed to the OMOP CDM

## **Secure data infrastructure**

The BIG-HEART cohort is stored within one of Estonia’s highest-security computing environments [6], featuring 13 000 CPU cores and 64 GPUs. Approved researchers access the environment remotely via a Virtual Private Network, using a browser-based virtual desktop interface. The system is fully isolated from the public internet. To ensure data protection, copying and pasting are disabled, and all user activity—including video recordings of analytic sessions—is logged for auditing. Data transfers are managed through secure S3 gateways, with every export subject to manual review and approval by a designated principal investigator.

## **Supplementary References**

1. Observational Health Data Sciences and Informatics (OHDSI). *OMOP Common Data Model.* <https://ohdsi.github.io/CommonDataModel/index.html> (12 February 2026, date last accessed).
2. Hripcsak G, Schuemie MJ, Madigan D *et al.* Drawing reproducible conclusions from observational clinical data with OHDSI. *Yearb Med Inform* 2021;**30**:283–289. <https://doi.org/10.1055/s-0041-1726481>
3. Observational Health Data Sciences and Informatics (OHDSI). *OMOP Common Data Model releases.* <https://github.com/OHDSI/CommonDataModel/releases> (12 February 2026, date last accessed).
4. Oja M, Tamm S, Mooses K *et al*. Transforming Estonian health data to the Observational Medical Outcomes Partnership (OMOP) Common Data Model: lessons learned. JAMIA Open 2023;**6**:ooad100. <https://doi.org/10.1093/jamiaopen/ooad100>
5. Observational Health Data Sciences and Informatics (OHDSI). *DataQualityDashboard*. <https://github.com/OHDSI/DataQualityDashboard> (12 February 2026, date last accessed).
6. HPC Public Documentation. *Sensitive data analysis platform SAPU*. <https://docs.hpc.ut.ee/public/services/SAPU/> (12 February 2026, date last accessed).
